# Engineering the Yeast *Yarrowia lipolytica* for Production of Polylactic Acid Homopolymer

**DOI:** 10.3389/fbioe.2020.00954

**Published:** 2020-10-22

**Authors:** Sophie Lajus, Simon Dusséaux, Jonathan Verbeke, Coraline Rigouin, Zhongpeng Guo, Maria Fatarova, Floriant Bellvert, Vinciane Borsenberger, Mélusine Bressy, Jean-Marc Nicaud, Alain Marty, Florence Bordes

**Affiliations:** ^1^TBI, CNRS, INRAE, INSA, Université de Toulouse, Toulouse, France; ^2^INRAE, AgroParisTech, Université Paris-Saclay, Micalis Institute, Jouy-en-Josas, France; ^3^Carbios, Biopôle Clermont Limagne, Saint-Beauzire, France

**Keywords:** *Yarrowia lipolytica*, polylactic acid, cellular compartments, PHA synthase, CoA analysis

## Abstract

Polylactic acid is a plastic polymer widely used in different applications from printing filaments for 3D printer to mulching films in agriculture, packaging materials, etc. Here, we report the production of poly-D-lactic acid (PDLA) in an engineered yeast strain of *Yarrowia lipolytica*. Firstly, the pathway for lactic acid consumption in this yeast was identified and interrupted. Then, the heterologous pathway for PDLA production, which contains a propionyl-CoA transferase (PCT) converting lactic acid into lactyl-CoA, and an evolved polyhydroxyalkanoic acid (PHA) synthase polymerizing lactyl-CoA, was introduced into the engineered strain. Among the different PCT proteins that were expressed in *Y. lipolytica*, the *Clostridium propionicum* PCT exhibited the highest efficiency in conversion of D-lactic acid to D-lactyl-CoA. We further evaluated the lactyl-CoA and PDLA productions by expressing this PCT and a variant of *Pseudomonas aeruginosa* PHA synthase at different subcellular localizations. The best PDLA production was obtained by expressing the PCT in the cytosol and the variant of PHA synthase in peroxisome. PDLA homopolymer accumulation in the cell reached 26 mg/g-DCW, and the molecular weights of the polymer (Mw = 50.5 × 10^3^ g/mol and Mn = 12.5 × 10^3^ g/mol) were among the highest reported for an *in vivo* production.

## Introduction

Today, most plastics are made from petroleum products and are highly persistent in the environment. With the growing awareness of the society concerns in the environment, the depletion of fossil resources and the increase in energy cost, the need for the development of sustainable production systems for polymer materials is more pressing than ever.

Polylactic acid produced from lactic acid, is expected to become both an alternative to petroleum-based plastic and a very important bio-based polymer because of its biocompatible properties and biodegradability in industrial composting. PLA can be easily processed by extrusion, injection, molding, blowing, dry-jet-wet spinning, etc., and allows a large range of applications, particularly for short-term uses (i.e., food packaging, bags, films, fibers, etc.) (Jamshidian et al., 2010). Due to their excellent biocompatibility and mechanical properties, PLA and its copolymers are becoming widely used in tissue engineering for function restoration of impaired tissues, in drug delivery systems, and in various medical implants (Jamshidian et al., 2010).

Lactic acid, the synthon of PLA, exists in two enantiomeric forms, D-lactic acid and L-lactic acid. Different types of polymers can be then synthetized from them, homopolymers, such as PLLA exclusively comprising L-lactic acid, PDLA only being made of D-lactic acid, and polymers consisting of both. Enantiomeric composition of the polymer modulates PLA mechanical properties, especially crystallinity percentage, glass transition and melting temperatures (Ingrao et al., 2015). Homopolymers are crystalline and exhibit a *T*_m_ around 180°C. The crystallinity and *T*_m_ of PLA polymers usually decrease as enantiopurity of the lactate units lowers. Interestingly, mixing PLLA and PDLA at a 1:1 ratio improves the polymer characteristic as it results in crystalline stereo-complex PLA with *T*_m_ 50°C higher than the one of the homopolymers (Masutani and Kimura, 2014).

To date, PLA is a widely used biobased polymer exclusively produced from the polymerization of lactic acid *via* a chemical process; for which the ring-opening polymerization of lactide (cyclic dimer of lactic acid) is the most effective method of synthesis in the industry. The polymerization process in itself raises environmental, economic and health concerns as it requires a high-energy input with extreme temperature and pressure conditions, and the use of metal catalyst that often produces harmful residues. Moreover, although lactic acid produced by bacterial fermentation from renewable resources can be used, further purification and concentration steps are needed to obtain a starting materials compatible with the subsequent chemical polymerization, thus increasing the chemical footprint of the whole process.

Developing a fully biological process thus offers an attractive perspective especially for environmental concerns. In the last decade, alternative biological *in vivo* processes have been developed to produce PDLA or co-polymer containing D-lactic acid (Taguchi et al., 2008; Jung et al., 2010; Yang et al., 2010, 2011; Jung and Lee, 2011). This eco-friendly process is composed of two enzymatic steps, first activation of D-lactic acid in D-lactyl-CoA catalyzed by a PCT, and then polymerization of the resulting D-lactyl-CoA by a PHA synthase (Supplementary Figure S1). The CoA activation of lactic acid is not naturally found in biological processes and no occurrence of this reaction has ever been described in the KEGG database. However, literature reports that PCT from some organisms are able to accept lactic acid as well as propionic acid as substrate (Yang et al., 2010; Lindenkamp et al., 2013). The second step, polymerization, takes advantage of the promiscuous activity of PHA synthase that naturally synthetizes polymers of hydroxy-fatty acids but that is also able to polymerize lactyl-CoA into PLA (instead of hydroxy-acyl-CoA into PHA). Taguchi et al. (2008) have engineered the *Pseudomonas* sp. 61-3 PHA synthase to polymerize together lactyl-CoA and 3HB-CoA (for 3-hydroxybutyrate) and demonstrated that *Escherichia coli* expressing the engineered PHA synthases produced poly-3HB-*co*-LA copolymer containing 6 mol% of lactic acid. Further improvement of the incorporation of lactic acid (up to 20 to 49 mol%) were achieved by co-expressing the evolved PHA synthases with the *C. propionicum* PCT (Yang et al., 2010). Interestingly, PDLA homopolymer were also detected although its accumulation was quite poor (2 and 7% of DCW, for Dry Cell Weight) (Yang et al., 2010). Nevertheless, the higher the lactate proportion was in the polymer, the lower was the polymer titer. Further metabolic engineering of the resultant *E. coli* strains was necessary to improve the PDLA accumulation up to 11% DCW (Jung et al., 2010).

In this study, we use the aerobic yeast *Yarrowia lipolytica* to produce PLA. Metabolic engineering of this robust yeast has already been reported for different applications such as efficient recombinant protein expression [lipases, cellulases, etc. (Guo et al., 2017; Madzak, 2018; Celińska and Nicaud, 2019)], aroma compound production (for review, see Coelho et al., 2010) or valuable fatty acid production (Beopoulos et al., 2014; Ledesma-Amaro and Nicaud, 2016; Rigouin et al., 2017). Interestingly, this yeast has also been proven to be convenient for the production of polyhydroxyalkanoates (PHA) (Haddouche et al., 2010, 2011; Gao et al., 2015). A recent work showed that PHA accumulation can reach up to 25% DCW (Rigouin et al., 2019), which demonstrates the potential use of this yeast for polymer accumulation.

Here, we attempted to implement a pathway enabling the yeast *Y. lipolytica* to produce a homopolymer only composed of D-lactic acid (PDLA). The first step was to prevent the consumption of the substrate by identifying enzymes that are involved in lactic acid consumption in *Y. lipolytica*. Afterward, we optimized the first step of the pathway by evaluating the ability of PCT proteins from several microorganisms to produce D-lactyl-CoA in *Y. lipolytica* and then, we investigate the influence of subcellular localization of the PCT on the production of this intermediate molecule. At last, PDLA production was quantified by expressing the PHA synthase targeted to different organelles.

## Materials and Methods

### Yeast Strains, Growth, and Culture Conditions for Strain Construction

All the plasmid constructions (Table 1) were made in the DH5 alpha strain (Promega, Madison, WI, United States). The *Y. lipolytica* strains used in this study were derived from the wild-type strain W29 (ATCC 20460) (see Table 2). The auxotrophic strain Po1d (Leu^–^, Ura^–^) has been described by Barth and Gaillardin (1996). Yl*CYB21* and Yl*DLD1* characterization was performed in the strain JMY2394 to facilitate both deletions and targeted integration at the zeta docking platform (*zeta*, *ku70*Δ) (Bordes et al., 2007; Verbeke et al., 2013). For clarity purpose, this strain will be named PK. PLA producing strains were derived from the strain JMY2159 (*MATA ura3-302 leu2-270 xpr2-322 pox1-6*Δ, *dga1*Δ, *lro1*Δ, *dga2*Δ, *fad2*Δ) (Beopoulos et al., 2014). The medium and growth conditions for *E. coli* were as described by Sambrook et al. (1989). Rich YPD medium (10 g/L yeast extract, 10 g/L peptone, 10 g/L glucose), minimal glucose medium (YNB containing 0.17% (w/v) yeast nitrogen base (without amino acids and ammonium sulfate, YNBww; Difco, Paris, France), 0.5% (*w/v*) NH_4_Cl, 0.1% (*w/v*) yeast extract (BD Bioscience, Sparks, MD, United States) and 50 mM phosphate buffer, pH 6.8 and minimal medium supplemented with casamino acids (YNBcasa, YNB supplemented with casamino acids; Difco, Paris, France) or uracil (YNBura) were prepared as described previously (Mlickova et al., 2004). Growth of the indicated strain was compared to the growth of the control strain (CS cont) on YNB medium containing different carbon sources: 10 g/L glucose (initial pH 6.8), or 10 g/L DL-lactic acid (racemic mixture), or 5 g/L D-lactic acid or 5 g/L L-lactic acid (initial pH 3.5 for all lactic acid cultures). All liquid cultures were done under agitation in Erlenmeyer baffled flasks with a ratio 1/5 between volume of culture and volume of flask.

**TABLE 1:** *E. coli* strains and plasmids.

**Strain (host strain)**	**Genotype or plasmid**	**References**
DH5a	F80d*lac*ZDm15, *rec*A1, *end*A1, *gyr*A96, *thi*-1, *hsd*R17 (r_k_-, m_k_+), *sup*E44, *rel*A1, *deo*R, D(*lac*ZYA*-arg*F)U169	Promega
JME547	pUB-Cre1 (*Cre ARS68 Hyg in*)	Fickers et al. (2003)
JME803	JMP62 URA3ex pTEF	Beopoulos et al. (2012)
JME804	JMP62 LEU2ex pTEF	Beopoulos et al. (2014)
JME2075	TopoPUT-DLD1	This work
JME2253	TopoPLT-CYB21	This work
JMY2316	JMP62 LEU2ex TEF-YlDLD1	This work
JMY2318	JMP62 LEU2ex TEF-YlCYB21	This work
ThEc_040	JMP62 URA3ex TEF-PaPHA synthase opt E130D, S325T, S477R, Q481M perox	This work
ThEc_055	JMP62 URA3ex TEF-PaPHA synthase opt E130D, S325T, S477R, Q481M cyto	This work
ThEc_039	JMP62 URA3ex TEF-PaPHA synthase opt E130D, S325T, S477R, Q481M mito	This work
ThEc_054	JMP62 LEU2ex TEF-CpPCT opt V193A cyto	This work
ThEc_021	JMP62 URA3ex TEF-EnPCT opt cyto	This work
ThEc_043	JMP62 URA3ex TEF-EcPCT opt cyto	This work
ThEc_045	JMP62 URA3ex TEF-RePCT opt cyto	This work
ThEc_018	JMP62 URA2ex TEF-YlACS2 opt cyto	This work
ThEc_016	JMP62 LEU2ex TEF-Cp-PCT opt V193A perox	This work
ThEc_038	JMP62 LEU2ex TEF-Cp-PCT opt V193A mito	This work
ThEc_041	JMP62 LEU2ex 4UAS-TEF-Cp-PCT opt V193A cyto	This work

**TABLE 2:** *Y. lipolytica* strains.

**Strain and used name**	**Parental strain**	**Genotype**	**References**
Po1d	/	*MATA ura*3-302 *leu*2-270 *xpr*2-322	Barth and Gaillardin (1996)
JMY2394 (PK)	Po1d	*ku70Δ, zeta*	Verbeke et al. (2013)
PK control	Po1d	*ku70::URA3ex, zeta, LEU2ex*	Verbeke et al. (2013)
PK dld1Δ	PK	*dld1::URA3ex, LEU2ex*	This work
PK dld1Δ + TEF-DLD1	PK	*dld1Δ, pTEF-YlDLD1-LEU2ex, URA3ex*	This work
PK cyb21Δ	PK	*cyb21::LEU2ex, URA3ex*	This work
PK cyb21Δ + TEF-CYB21	PK	*cyb21*Δ, *pTEF-YlCYB21-LEU2ex, URA3ex*	This work
JMY2159	Po1d	*pox1-6Δ, dga1Δ, lro1Δ, dga2Δ, fad2Δ*	Beopoulos et al. (2014)
CS (for chassis strain)	JMY2159	*dld1Δ*	This work
CS cont	CS	*dld1Δ, URA3ex, LEU2ex*	This work
CS TEF-CpPCTc	CS	*dld1Δ, TEF-CpPCT opt V193A cyto-LEU2ex, URA3ex*	This work
CS TEF-CpPCTp	CS	*dld1Δ, TEF-CpPCT opt V193A pero-LEU2ex, URA3ex*	This work
CS TEF-CpPCTm	CS	*dld1Δ, TEF-CpPCT opt V193A mito-LEU2ex, URA3ex*	This work
CS HTEF-Cp PCTc	CS	*dld1Δ, HTEF-CpPCT opt V193A cyto-LEU2ex, URA3ex*	This work
CS TEF-EcPCTc	CS	*dld1Δ, TEF-EcPCT opt cyto-URA3ex, LEU2ex*	This work
CS TEF-RePCTc	CS	*dld1Δ, TEF-RePCT opt cyto-URA3ex, LEU2ex*	This work
CS TEF-EnPCTc	CS	*dld1Δ, TEF-EnPCT opt cyto-URA3ex, LEU2ex*	This work
CS TEF-YlACS2c	CS	*dld1Δ, TEF-YlACS2 opt cyto-URA3ex, LEU2ex*	This work
CS TEF-CpPCTc + TEF-PaPHAp	CS	*dld1Δ, TEF-CpPCT opt V193A cyto-LEU2ex, TEF-PaPHAC opt E130D, S325T, S477R, Q481M perox-URA3ex*	This work
CS TEF-CpPCTc + TEF-PaPHAm	CS	*dld1Δ, TEF-CpPCT opt V193A cyto-LEU2ex, TEF-PaPHAC opt E130D, S325T, S477R, Q481M mito-URA3ex*	This work
CS TEF-CpPCTc + TEF-PaPHAc	CS	*dld1Δ, TEF-CpPCT opt V193A cyto-LEU2ex, TEF-PaPHAC opt E130D, S325T, S477R, Q481M cyto-URA3ex*	This work
CS HTEF-CpPCTc + TEF-PaPHAp	CS	*dld1Δ, HTEF-CpPCT opt V193A cyto-LEU2ex, TEF-PaPHAC opt E130D, S325T, S477R, Q481M perox-URA3ex*	This work
CS HTEF-CpPCTc + HTEF-PaPHAp	CS	*dld1Δ, HTEF-CpPCT opt V193A cyto-LEU2ex, HTEF-PaPHAC opt E130D, S325T, S477R, Q481M perox-URA3ex*	This work

### *In silico* Sequence Analysis

Gene and protein sequences were obtained from NCBI,^1^ UniprotKB^2^ and the yeast genomic database Génolevures.^3^ Peptidic sequence alignments were realized by Clustal Omega at the EMBL-EBI server.^4^ LDH characteristic protein domains were searched with Pfam server^5^ (El-Gebali et al., 2019) that include an Interproscan analysis (Finn et al., 2017).

Target signal predictions were realized at the CBS predictions servers^6^ and Mitoprot.^7^

### General Genetic Techniques

Restriction enzymes were obtained from New England Biolabs (Evry, France). Genomic DNA from yeast transformants was obtained as described by Querol et al. (1992). An Applied Biosystem 2720 thermal cycler (Thermo Fisher Scientific, Courtaboeuf, France) with both *Taq* (Takara, Shiga, Japan) and *Pyrobest* (Takara, Shiga, Japan) DNA polymerase was used for PCR amplification. Amplified fragments were purified with the QIAgen Purification Kit (Qiagen, Hilden, Germany) and DNA fragments were recovered from agarose gels with the QIAquick Gel Extraction Kit (Qiagen). The yeast cells were transformed by the lithium acetate method (Barth and Gaillardin, 1996) or using Frozen-EZ Transformation kit (Zymo Research, Irvine, CA, United States).

### Construction of Disrupted Strains Followed by Marker Excision

The deletion cassettes were generated by PCR amplification as described by Fickers et al. (2003). Briefly, the promoter (P) and terminator regions (T) were amplified from genomic DNA and fused by PCR with an I-*Sce*I restriction site at the junction. The PT resulting cassettes were then inserted into the PCR4RBlunt-TOPO vector (Life Technologies, Carlsbad, CA, United States) and the auxotrophic marker (either URA3ex or LEU2ex amplified by PCR) was then inserted into the vectors using I-*Sce*I restriction site to generate the corresponding (respectively PUT or PLT) vectors (Table 1). The PUT and PLT disruption cassettes were transformed into *Y. lipolytica*. Transformants were selected on appropriated selective media YNBcasa or YNBura, respectively, or YNB when transformants were prototroph. The corresponding ver1 and ver2 primers (Supplementary Table S1) were used to check gene disruption by PCR amplification of the genomic loci. Marker rescue was performed after transformation with the replicative plasmid pUB4-CreI as described by Fickers et al. (2003). The list of the primers for cassette design and strain verification is given in Supplementary Table S1.

### Cloning and Expression of the Individual Genes of Interest Under Control of the TEF and HTEF Constitutive Promoters

Yl*DLD*1 and Yl*CYB*21 genes were respectively amplified from genomic DNA of *Y. lipolytica* with primer couples ylDLD1for/ylDLD1rev and ylCYB21for/ylCYB21rev (Supplementary Table S1). After digestion by *Bam*HI and *Avr*II, the DNA fragments were inserted into the corresponding sites of the expression vector, deriving from JMP62 (Nicaud et al., 2002) under the control of TEF constitutive promoter (Müller et al., 1998).

The genes encoding CoA transferase from *E. coli* (EcPCT; GenBank accession number: AFJ29290.1), PCT from *Emiricella nidulans* FGSC A4 (EnPCT; GenBank accession number: EAA58342.1), PCT from *Ralstonia eutropha* H16 (RePCT; GenBank accession number: CAJ93797.1), variant V193A of PCT from *Clostridium propionicum* (CpPCT; GenBank accession number: CAB77207.1, Yang et al., 2010), and *ACS2*, encoding acetyl-CoA synthase from *Y. lipolytica* (YlACS2; accession number: XP_505057.1), were synthesized by GenScript (Hong Kong, China) with codon optimization based on the codon bias of *Y. lipolytica* (sequences in Supplementary Information), and cloned under the expression promoter TEF or hybrid promoter HTEF (Nicaud et al., 2002; Dulermo et al., 2017).

The plasmid allowing the expression of variants of PHA synthase from *Pseudomonas aeruginosa* corresponds to the JMP62-URA3-PHA plasmid (Haddouche et al., 2010) in which the POX promoter has been replaced by the TEF promoter by a *Cla*I-*Bam*HI cloning.

The variants of the PHA synthase were constructed by site directed mutagenesis using the QuikChange II Site-Directed Mutagenesis Kit (Agilent, Santa Clara, CA, United States), and primers are listed in Supplementary Table S2. All the sequences were checked by Sanger sequencing (Eurofins Genomics, Les Ulis, France).

After construction, all the plasmids were linearized by *Not*I and used to transform *Y. lipolytica*. Yeast transformants were selected by auxotrophy on the adequate minimal medium and the presence of the genes of interest was verified by PCR.

Proteins were targeted to different subcellular compartments using specific targeting signals. For peroxisome targeting, PTS1 tag, which corresponds to the last 14 amino acids of the *Y. lipolytica* isocitrate dehydrogenase (Uniprot accession number: P41555) was introduced at the C-terminal part of the protein of interest (Smith et al., 2000). For mitochondria targeting, MTS tag corresponds to the first 26 amino acids of the *Y. lipolytica* mitochondrial malic enzyme (Uniprot accession number: Q6C5F0) and was fused at the N-terminal part of the protein of interest.

### Culture Conditions for PLA Production

Yeast cells were first grown on rich YPD medium at 28°C overnight. Cells were then harvested by centrifugation to remove medium and resuspended in minimal medium YNBcasa with 30 g/L glucose and 10 g/L of racemic lactic acid (L enantiomer used as carbon source and D enantiomer used as synthon for PDLA production) with an initial OD_600__nm_ around 0.5 measured with a DR 3900 spectrophotometer (Hach Lange, Loveland, CO, United States). The cultures were grown at 28°C, with an agitation of 100 rpm for 5 days. Cells were then harvested by centrifugation, washed twice with water, and the cell pellet was kept at −80°C until further analysis. Culture media were filtered through a 0.4 μm filter and kept at −20°C until analyzed.

### Substrate Analysis

Glucose concentration was determined using a YSI 2900 analyzer (System C industrie, St Paul Trois Chateaux, France) or by high-performance liquid chromatography using Thermo Fisher Scientific system equipped with a RI detector and a Biorad column (Aminex HPX-87H column 300 × 7.8 mm, Marnes la Coquette, France) at 40°C using 5 mM H_2_SO_4_ as mobile phase at 0.4 mL/min.

Lactate concentration was determined by high-performance liquid chromatography in the same conditions described for glucose quantification. The enantiomeric composition of lactic acid was determined by high-performance liquid chromatography using Thermo Fisher Scientific system equipped with a UV detector at 254 nm and Chirex 3126 (D)-penicillamine 150 × 4.6 mm column (Phenomenex, Le Pecq, France) at 22°C using 2 mM CuSO_4_, 15% methanol (v/v) as the mobile phase at 1 mL/min.

### Microscopy

To observe PLA accumulation, cells were grown as described in section “Culture Conditions for PLA Production” and washed twice with water. Pellets were then resuspended in water and PLA accumulation was immediately visualized in the cells using BODIPY 493/503 dye (Thermo Fisher Scientific) diluted at 1 μg/mL and imaged using Eclipse Ni-E epifluorescence microscope (Nikon France S.A, Champigny sur Marne, France).

### Lactyl-CoA Extraction and Determination

Yeast strains were grown in minimal medium YNBcasa with 30 g/L glucose and 10 g/L of racemic lactic acid. For PCT enantiospecificity analysis, instead of 10 g/L of racemic lactic acid solution, a mixture of 5 g/L of unlabeled L-lactic acid and 5 g/L of labeled D-lactic acid-3-^13^C (Sigma–Aldrich, St. Quentin Fallavier, France) was used (named also (^13^C)-D-lactic acid in this paper). At indicated time, cells were collected, separated from medium by fast filtration, and washed with 10-fold diluted medium. The filter was then placed in liquid nitrogen for rapid freezing and kept at −80°C before extraction. Then, the filter was placed in chilled extraction solution (80% methanol, 20% water with 125 mM formic acid) and subject to 3 cycles of 10 second vortex and 10 second sonication. Samples were collected, dried, and resuspended in analysis buffer (2% methanol, 98% water with 25 mM ammonium formate) by vortexing and sonication.

Yeast extracts were analyzed by UHPLC-FTMS (LTQ Orbitrap velos, Thermo Fisher Scientific) for lactyl-CoA quantification. Samples were kept at 4°C in the autosampler. A reversed phase C-18 column (Phenomenex Kinetex, 100 mm × 3 mm, particle size 1.7 μm, guard column SecurityGuard Ultra) was used for the separation (at 40°C) with a gradient of 50 mM ammonium formate adjusted to pH 8.10 with ammonium hydroxide (solvent A) and methanol (solvent B). The flow rate was set to 0.4 mL/min and the multi-step linear gradient was: 2% B at 0 min, 2% B at 2 min, 25% B at 23 min, 95% B at 23.1 min, 95% B at 28 min, 2% B at 28.1 min, 2% B at 36 min. The injection volume was 5 μL (full loop mode). Mass spectra were acquired with FTMS in positive mode with electrospray ionization at resolution R = 60,000 (at *m/z* 400) and recorded for the range of *m/z* 750-1500. Source temperature was 250°C, capillary temperature was 275°C, sheath gas at 50 AU, auxiliary gas at 10 AU, S lens level at 70%, and source voltage 4 kV. Qualitative and quantitative analyses were performed with Trace Finder software (Thermo Fisher Scientific). Identification and quantification of lactyl-CoA and lactyl-3-^13^C-CoA were determined by extracting and integrating their exact mass (5 ppm) with Tracefinder^®^ software (Thermo Fisher Scientific). The correction of the area of lactyl-3-^13^C-CoA for naturally occurring isotopes from lactyl-CoA was performed with IsoCor^®^ adapted for high resolution mass spectrometry (Millard et al., 2012; Heuillet et al., 2018).

### PLA Extraction and Analysis

About 1.5 g of lyophilized cells were resuspended in 15 mL of 100 mM Tris, pH 8 added with 0.5 mg/mL zymolyase and incubated at 25°C overnight. Cell suspensions were frozen at −80°C and freeze dried until polymer extraction. Produced polymer was extracted using a Soxhlet apparatus with chloroform. To do so, about 1.5 g of dried cells were used and the chamber of the Soxhlet apparatus was filled 10 times with solvent, then solvent, and extracted materials were collected.

When necessary, PLA polymer was precipitated to remove residual fatty acids as described by Nomura et al. (2004). Briefly, PLA was precipitated by addition of ten volumes of cyclohexane to the solution of PLA in chloroform and collected by centrifugation.

Polymer composition was determined by NMR on a Bruker Avance II 500 spectrometer (Bruker Wissembourg, France). The cells extracts were thoroughly dried, prior to being diluted in CDCl_3_ containing 1% TMS (internal standard), and transferred to 5 mm NMR tubes. NMR spectra were recorded at 298 K. Each NMR spectrum was acquired using an excitation flip angle of 30° at a radiofrequency field of 29.7 kHz, a relaxation delay of 10 s and 2 dummy scans. For each experiment, 16 scans were performed with a repetition delay of 6.5 s. PLA concentrations were determined by integration of the specific quadruplet signal at 5.19 ppm.

Molecular weight averages and dispersity of the polymer were determined by gel permeation chromatography (GPC) at 20°C using a Shimadzu system (Marne la Vallée, France) equipped with Wyatt detectors (MALLS, Dawn Heleos-II, 18 angles and refractometer, Optilab T-rEX, Toulouse, France) with two Agilent columns (PLGel 5um MIXED-C 300 × 7.5 mm). The elution solvent used was dichloromethane. Samples were resuspended in dichloromethane and filtered through a 0.4 μm filter. The weight averages and dispersity were calculated using dn/dc value determined for these conditions and equal to 0.0296.

Polylactic acid composition was analyzed according to Faisal et al. (2007). Briefly, PLA hydrolysis was conducted at 180°C and 100 bars, in distilled water for 50 min using ASE system (Thermo Scientific, Villebon-sur-Yvette, France). The hydrolysate was collected and analyzed as described in section “Substrate Analysis” for enantiomeric composition of lactic acid.

## Results

### Lactate Uptake: *Y. lipolytica*’s Lactate Dehydrogenases

#### *In silico* Identification

To maximize the pool of substrate for PLA production, we investigated lactic acid catabolism in the yeast *Y. lipolytica*. Mansour et al. (2008) showed that this yeast is capable of consuming lactic acid but the proteins responsible for its consumption remained unknown. In *Saccharomyces cerevisiae*, two classes of enzyme are responsible for the conversion of lactic acid to pyruvate: LDH, that are encoded by DLD genes, and lactate cytochrome oxydoreductase (encoded by CYB2 gene) (Ookubo et al., 2008). Therefore, a BLAST research on *Y. lipolytica* genome was carried out on the basis of these two classes of enzymes, revealing 4 genes namely Yl*DLD1*, Yl*DLD2*, Yl*CYB21* and Yl*CYB22* (YALI0E03212, YALI0C06446, YALI0D12661, and YALI0E21307, respectively) encoding putative lactate degradation enzymes. Analysis of their protein sequences using Mitoprot website showed that YlDld1p and YlDld2p may contain a N-terminal mitochondrial target signal (respective scores of 0.9998 and 0.9962), suggesting that these LDH are targeted to mitochondria. The presence of peroxisomal target sequences PTS1 (SKL for YlCyb22p and VKL for YlCyb21p) on both Yl*Cyb* sequences suggested the peroxisome as their subcellular location. Pfam and Interproscan website analyses indicated that YlDld1p and YlDld2p present a FAD_binding_4 domain (PF01565 family), suggesting that they may exhibit specific activity toward D-lactic acid whereas YlCyb21p and YlCyb22p present a Cyt-b5 domain (PF00173 family), indicating that they may have specific activity for L-lactic acid.

#### Disruption and Complementation of the *Y. lipolytica* Lactate Degradation Enzymes

**FIGURE 1:**
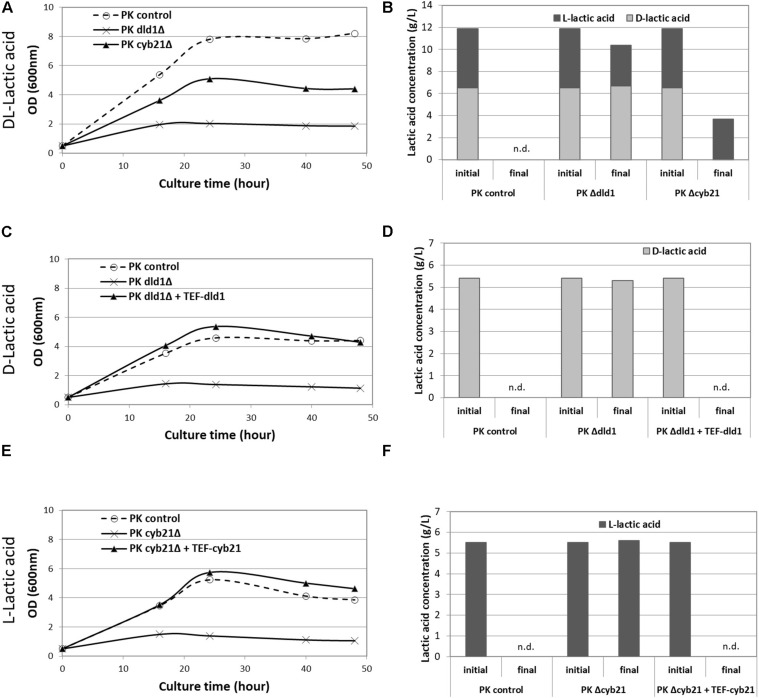
Growth curves of strains with lactate dehydrogenase deletion and lactic acid consumption. Growth curves of PK control strain, PK dld1Δ and PK cyb21Δ strains with DL-lactic acid as carbon source **(A)**, and their lactic acid consumption **(B)**; PK control strain, PK dld1Δ and PK dld1Δ + TEF-DLD1 strains on D-lactic acid **(C)** and their D-lactic acid consumption **(D)**; PK control strain, PK cyb21Δ and PK cyb21Δ + TEF-CYB21 strains on L-lactic acid **(E)** and their L-lactic acid consumption **(F)**. Data presented are representative of at least three independent experiments. n.d., not detected.

In order to elucidate the role of the identified enzymes, we first performed the deletions of those genes in the Po1d zeta *Ku70*Δ strain, designated as PK thereafter (Verbeke et al., 2013). This strain shows a better rate of homologous recombination, which facilitates downstream deletions. Transformants were tested for their ability to grow on different carbon sources: glucose, D- or L-lactic acid or a racemic mixture of the latter (Figure 1). All tested strains grew equally on glucose and Yl*CYB22* and Yl*DLD2* deletions did not affect the growth of strains on any lactic acid substrate, compared with the control strain, suggesting that these genes were not implicated in lactic acid metabolism (data not shown). On the contrary, deletion of Yl*CYB21* and Yl*DLD1* genes partially abolished the growth of the corresponding strains (PK c*yb21*Δ and PK *dld1*Δ) on a racemic mixture of L- and D-lactic acid (Figure 1A). Consistently, no consumption of L- or D-lactic acid was observed for those corresponding strains (Figure 1B). Of note, the growth of PK *dld1*Δ strain was highly impeded (Figure 1A), probably due to its limited L-lactic acid consumption (Figure 1B). However, as expected, this strain was not capable to consume D-lactic acid (Figure 1B).

To confirm the observed phenotypes, we overexpressed the deleted genes under the control of the constitutive promoter TEF in a rescue experiment. As shown in Figures 1C,D, the overexpression of Yl*DLD1* gene in PK *dld1*Δ strain completely restored the growth and D-lactic acid consumption. Similarly, overexpression of Yl*CYB21* gene in the deleted PK *cyb21*Δ strain restored the growth of the resulting strain on L-lactic acid (Figures 1E,F). Taken together, our results clearly illustrated that in *Y. lipolytica*, Yl*CYB21* and Yl*DLD1* genes encode for LDH, specific for L- and D-lactic acid, respectively.

#### Chassis Strain Construction

The JMY2159 strain (*MATA ura3-302 leu2-270 xpr2-322 pox1-6*Δ, *dga1*Δ, *lro1*Δ, *dga2*Δ, *fad2*Δ) (Beopoulos et al., 2014), constructed for another purpose, combines different modifications suitable for PDLA production: (1) the deletion of the six genes encoding the acyl-CoA dehydrogenase (*POX* genes) abolishes the fatty acid degradation in the peroxisome into 3-hydroxy-acyl-CoA, the natural substrate of PHA synthase: it will allow the production of a homopolymer of D-lactic acid; (2) the deletion of the 3 acyl-transferase genes (*DGA1*, *DGA2* and *LRO1*) prevents the formation of triacylglycerols and their accumulation in lipid bodies: it will allow visualization of PLA production using the lipophilic BODIPY fluorescent dye staining without interference of fluorescence associated with lipid bodies; (3) the Δ12 desaturase (*FAD2*) has also been removed from this strain but the deletion was not reverted since it does not interfere with PLA metabolic pathway. To perform our study, we further deleted Yl*DLD1* gene and, after prototrophy restoration of the strain, we obtained the chassis strain that was, as expected, unable to grow on D-lactic acid and presented the following genotype *MATA ura3-302 leu2-270 xpr2-322 pox1-6*Δ, *dga1*Δ, *lro1*Δ, *dga2*Δ, *fad2*Δ, *dld1*Δ. All the following constructions were performed in this chassis strain, subsequently named CS for clarity purposes (Table 2).

### Lactyl-CoA Production Catalyzed by PCT

#### Expression of Different PCT Proteins

To be used as substrate by the PHA synthase, lactic acid has first to be activated into lactyl-CoA. We selected different PCT enzymes for this reaction: (1) PCT from *C. propionicum* (CpPCT) and PCT from *R. eutropha* (RePCT) that have been shown to be able to use lactic acid in addition to propionate as substrate (Yang et al., 2010; Lindenkamp et al., 2013), (2) a CoA transferase from *E. coli* (EcPCT) described to be active on short chain length substrates (Rangarajan et al., 2005), (3) a PCT from *E. nidulans* FGSC A4 (EnPCT) (Brock and Buckel, 2004), and (4) a potential acetyl-coenzyme A synthase from *Y. lipolytica* (YlACS2 encoded by YALI0F05962g), which presents 28% of identity with the PCT from *C. propionicum*. As a first approach, all these enzymes were expressed in the chassis strain under the control of the constitutive TEF promoter and without addition of any targeting signal, meaning that proteins were expected to be expressed in the cytosol. Throughout the text, cytoplasmic expression is indicated by a “c” at the end of the protein name.

**FIGURE 2:**
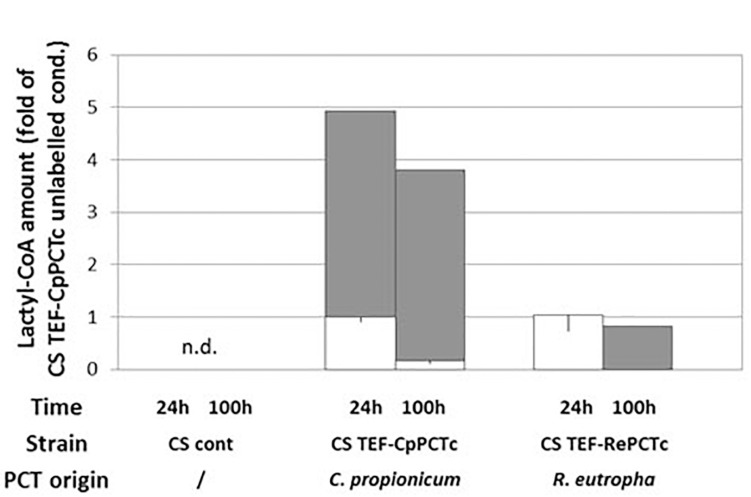
Quantification of D- and L-lactyl-CoA produced in strain expressing PCT protein from different organisms. Strains were grown for 24 h and 100 h on minimum medium containing a mixture of 5 g/L of unlabeled L-lactic acid and 5 g/L of labeled D-lactic acid 3-^13^C. After a normalization, results were expressed as fold of CS + TEF-CpPCTc strain unlabeled result at 24 h. White bars: unlabeled compound, gray bars: ^13^C-labeled compound. For clarity purposes, only half error bars have been represented; to the bottom for unlabeled compound and to the top for labeled one. *N* = 3 independent experiments. n.d., not detected.

To study the functionality of the above enzymes to produce lactyl-CoA and their putative enantio-specificity for lactic acid substrate, *Y. lipolytica* strains expressing individual CoA transferase protein were grown on minimum medium supplemented with glucose, L-lactic acid and (^13^C)- D-lactic acid. The utilization of labeled (^13^C)-D-lactic acid, allowed to distinguish by mass spectrometry labeled (^13^C)-D-lactyl-CoA from unlabeled L-lactyl-CoA. After 24 h of cultivation, similar cellular levels of acetyl-CoA were detected for all strains (Supplementary Figure S2). As shown in Figure 2, only the expression of CpPCTc or RePCTc enzymes allowed the production of lactyl-CoA which is not detectable in CS control. Lactyl-CoA was not detectable in strains expressing EcPCT, EnPCT and YlACS2 (data not shown). Regarding the enantiospecificity, the strain expressing CpPCTc produced about 80% of labeled D-lactyl-CoA at 24 h and 95% after 100 h of culture, suggesting that this enzyme is specific for D-lactic acid (Figure 2). Surprisingly, for the strain expressing RePCTc, production of L-lactyl-CoA was detected after 24 h but no labeled D-lactyl-CoA was detected. However, after 100 h of cultivation, this strain also produced labeled D-lactyl-CoA, but the production was only about 25% of the production obtained by the strain expressing CpPCTc. Therefore, CpPCT was chosen for further engineering of *Y. lipolytica* for the production of PDLA.

#### Subcellular Localization of PCT Protein

**FIGURE 3:**
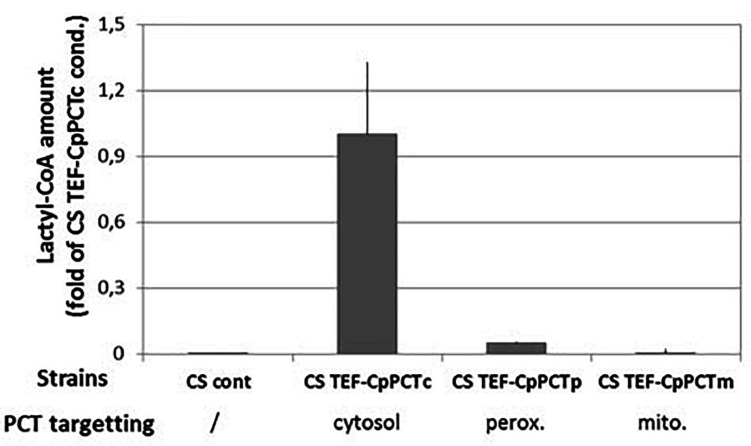
Quantification of lactyl-CoA produced in strain expressing PCT protein from *C. propionicum* targeted to different subcellular compartments. Strains were grown for 24 h on minimum medium containing a mixture of 10 g/L of lactic acid. After a normalization, results were expressed as fold of CS TEF-CpPCTc strain result. For clarity purposes, only half error bars have been represented. *N* = 3 independent experiments.

*In silico* analysis suggested that YlDld1 protein is likely to be targeted to mitochondria, indicating that the metabolism of D-lactic acid may be favored in this specific compartment. In order to study the impact of PCT localization within the cell on lactyl-CoA production, cytosolic expression (CpPCTc) was compared with mitochondrial (CpPCTm) or peroxisomal expression (CpPCTp), as production of PHA polymers has already been described in this compartment (Haddouche et al., 2010). We then measured lactyl-CoA production by these strains. As we showed that CpPCT was specific for D-lactic acid, cultures were performed using unlabeled D-lactic acid. After 24 h of cultivation, quantification of CoA compounds was performed. The analysis showed that acetyl-CoA amounts were equivalent in all the strains (Supplementary Figure S3). Nevertheless, the amount of lactyl-CoA was highly affected by the subcellular localization of the CpPCT. Indeed, lactyl-CoA was almost undetectable when CpPCT was targeted to the mitochondrial matrix (Figure 3). When CpPCT was addressed to the peroxisomes, lactyl-CoA production was dramatically decreased (about 10-fold) compared to cytosolic expression (Figure 3). Consequently, CpPCT was expressed as a cytosolic protein in the following experiments to optimize the first step of PDLA production.

### PLA Production and Characterizations of the Polymer

#### Expression of PHA Synthase

**FIGURE 4:**
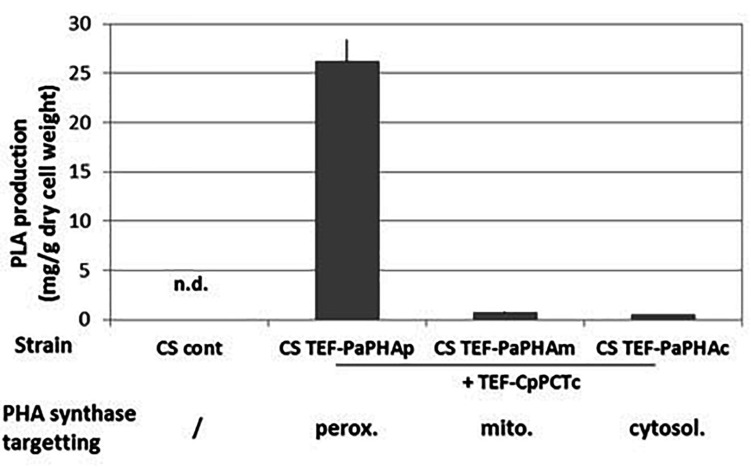
PDLA quantification in strains expressing PHA polymerase targeted to different subcellular compartments. Strains expressing cytosolic PCT from *C. propionicum* and PHA synthase from *P. aeruginosa* targeted to indicated compartment were grown for 5 days on minimum medium containing a mixture of 10 g/L of lactic acid. After extraction, PLA quantification was measured by NMR using PLA specific peaks. *N* = 3 minimum independent experiments. n.d., not detected.

The second step for PLA production is the polymerization of D-lactyl-CoA into PDLA by the PHA synthase (Supplementary Figure S1). The ability of the yeast *Y. lipolytica* to produce PHA polymers in peroxisomes has been demonstrated previously by targeting a PHA synthase from *P. aeruginosa* to this compartment (Haddouche et al., 2010, 2011; Gao et al., 2015; Rigouin et al., 2019). The ability of the PHA synthase from *Pseudomonas* species to produce polymer containing lactic acid was already demonstrated in bacteria (for example, see Taguchi et al., 2008). Taguchi et al. (2008) described many different variants of the PHA synthase with an improved ability to use short synthons, such as lactyl-CoA. In the present work, we co-expressed CpPCTc with either the *P. aeruginosa* wild-type PHA synthase or with the quadruple variant (E130D, S325T, S477R, and Q481M) which has been described to allow the production of PDLA in engineered bacteria (Jung et al., 2010). After 5 days of culture and extraction, PDLA was quantified by NMR analysis. Our results showed that expressing the wild-type PHA synthase did not lead to any PDLA polymer production neither expressed in the cytosol nor targeted to the peroxisome (data not shown). When expressing the *P. aeruginosa* quadruple PHA synthase variant either in the cytosol or in the mitochondria very low level of PDLA was detected (Figure 4). For both cytoplasmic and mitochondrial targeting, the low levels of PDLA accumulation might result from low activity in these compartments or low substrate concentration. However, when the PHA synthase variant was targeted into peroxisomes, PDLA was successfully accumulated (Figure 4). Later, PaPHAp will refer to the quadruple variant targeted into the peroxisomes.

To confirm this result, we followed PDLA accumulation by fluorescence microscopy using BODIPY dye (Supplementary Figure S4). Images clearly showed no PDLA accumulation in the CS strain. However, in the strain CS TEF-CpPCTc + TEF-PaPHAp, PDLA production was detected after 2 days and this production increased during the three following days. These results demonstrate that D-lactyl-CoA produced in the cytosol is likely transported into peroxisomes for an efficient polymerization by PHA synthase quadruple variant. After 5 days, the biomass reached 6.1 g/L and the accumulation of PLA reached 26.2 ± 2.3 mg/g DCW, corresponding to a titer of 158.5 mg/L. However, optimization of the production was not the purpose of this study and these data are only based on samples obtained after 5 days of culture in baffled flasks.

**FIGURE 5:**
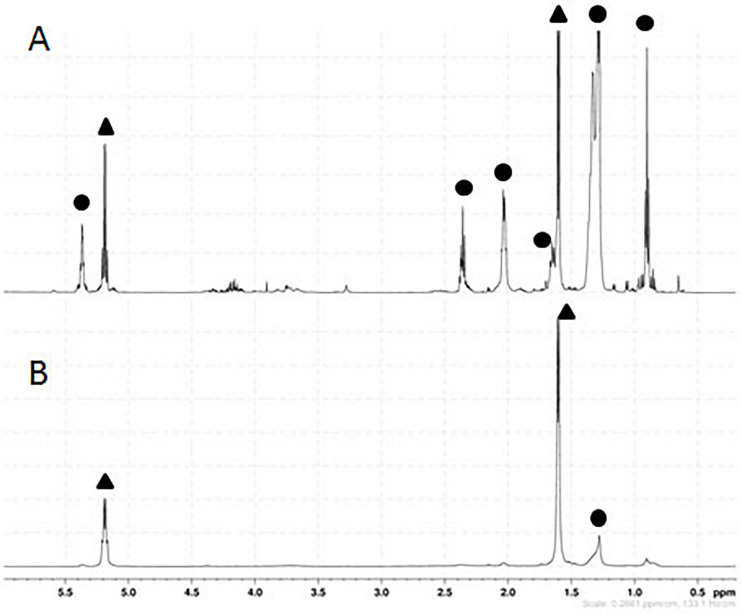
Proton nuclear magnetic resonance (1H-NMR) spectra of PLA. PLA was extracted from lyophilized culture of CS TEF-CpPCTc + TEF-PaPHAp strain using chloroform soxhlet **(A)** and then purified **(B)**. Signals pertaining to PLA are designated by a black triangle, whilst signals belonging to free fatty acids from the cellular extraction are indicated by a black circle. These experiments are representative of three independent experiments.

**FIGURE 6:**
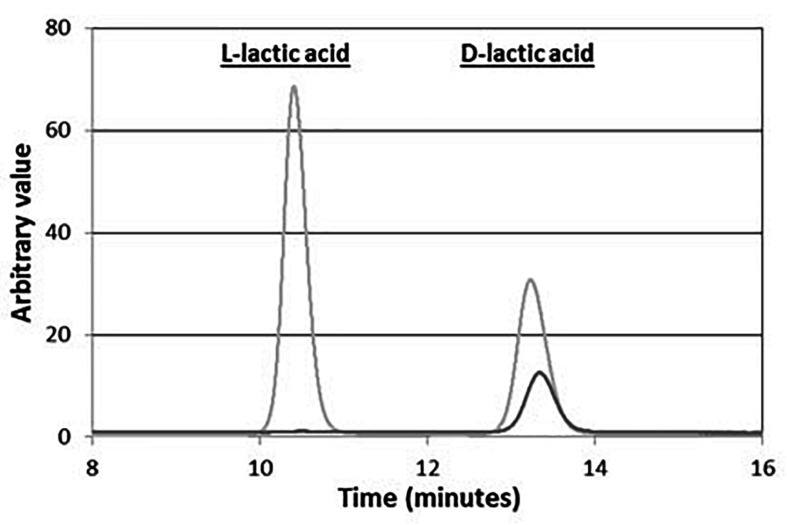
Composition in lactic acid of PLA produced *in vivo* in CS TEF-CpPCTc + TEF-PaPHAp yeast strain after hydrolysis. PLA was extracted from CS + TEF-CpPCTc + TEF-PaPHAp strain after 5-day cultivation, then purified and hydrolyzed. The extracted solution was then analyzed by HPLC. This experiment is representative of three independent experiments.

As shown in Figure 5, the ^1^H-NMR spectra of the polymer produced with this strain displayed specific quadruplet of PLA homopolymers (copolymers produce an unresolvable multiplet), but also signals of free fatty acids (Figure 5A). Furthermore, the double doublets (type AB) between 2.66 and 2.45 ppm, which are characteristic of 3-hydroxy-fatty acids were not observed in the spectra indicating that the PLA polymer was solely constituted of lactic acid units. After a step of purification by cyclohexane precipitation, ^1^H-NMR of the purified PLA showed that fatty acids contaminants were successfully removed (Figure 5B). To further determine the lactic acid enantiomeric composition of the polymer, it was subjected to total hydrolysis (Faisal et al., 2007), and the hydrolysate was analyzed by HPLC using an enantiospecific lactic acid column. Only D-lactic acid could be detected (Figure 6). The combined analysis done by NMR and by HPLC give evidence that the polymer produced *in vivo* is an enantiopure PDLA homopolymer devoid of any 3-hydroxy-fatty acid. Additionally, the polymer size was determined using GPC and we found that PDLA molecular weights (Mw) reached 50.5 × 10^3^ g/mol and dispersity of 4.05.

#### Optimization of PDLA Production

In order to determine the best production conditions, we assessed the impact of the substrate supply (D-lactic acid) on PDLA production in the CS TEF-CpPCTc + TEF-PaPHAp strain. For this purpose, cells were grown in minimum medium supplemented with glucose and L-lactic acid at 5 g/L with D-lactic acid concentration ranging from 0.5 g/L to 10 g/L. We observed that PDLA production increased when initial D-lactic acid concentration varied from 0.5 g/L to 5 g/L then reached a plateau (Supplementary Figure S5). D-Lactic acid was quantified in the medium at the end of the cultures and no culture was depleted in lactic acid (data not shown). It was worth noting that in all cases, D-lactic acid introduced was in excess in the media regarding PDLA production. These results demonstrate that when initial D-lactic acid concentration is equal to or above 5 g/L, the substrate supply is not bottlenecking the pathway.

**FIGURE 7:**
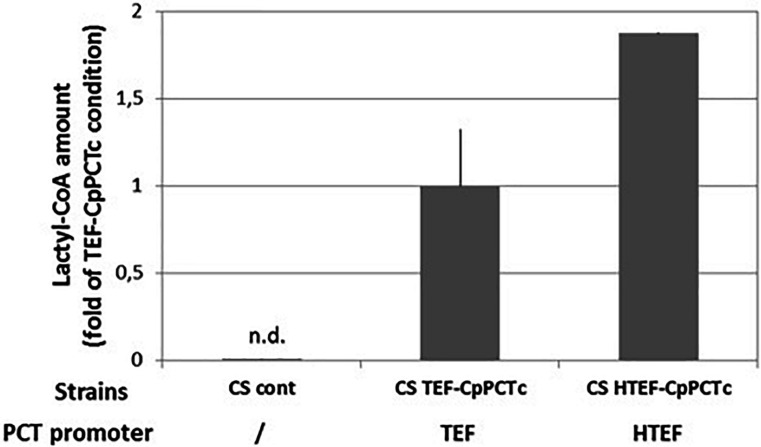
Quantification of lactyl-CoA produced in strain expressing *C. propionicum* cytosolic PCT protein under the control of different promoters. Strains were grown for 24 h on minimum medium containing a mixture of 10 g/L of lactic acid. After a normalization, results were expressed as fold of CS TEF-CpPCTc strain result. For clarity purposes, only half error bars have been represented. *N* = 3 independent experiments.

To improve PDLA production, we increased PCT and PHA synthase expression level using a hybrid TEF promoter (HTEF) which has showed to increase protein expression (Dulermo et al., 2017). The promoter change did not affect acetyl-CoA level (CS HTEF-CpPCTc) but did increase two-fold the cellular lactyl-CoA content (Figure 7 and Supplementary Figure S6).

As using HTEF promoter to control CpPCT expression has a positive impact on lactyl-CoA accumulation, new strains were constructed expressing CpPCTc under HTEF control and PHA synthase variant under either the control of the TEF or the HTEF promoter. A first PDLA quantification was determined by strains grown in the conditions optimized for PLA production. Interestingly, these preliminary results showed the improvement of PDLA production to 30.6 mg/g _DCW_ for HTEF-CpCPTc + TEF-PaPHAp and up to 34.8 mg/g _DCW_ for HTEF-CpCPTc + HTEF-PaPHAp corresponding to a 32% improvement in PDLA production (Supplementary Figure S7). However, using HTEF promoter to control CpCPTc (HTEF/TEF) or both genes (HTEF/HTEF), the Mw of the polymers observed decreased (from 50.5 × 10^3^g/mol to 30.3 and 22.5 × 10^3^ g/mol, respectively).

## Discussion

Although microbial PHA production has been extensively described in a variety of organisms that could accumulate impressive quantities of biopolymer (>80% DCW) (Choi et al., 2020), PLA biosynthesis has been only reported in *E. coli* strains so far. Despite the initial PLA accumulation in the engineered *E. coli* was very low (of only 0.5% DCW), further improvement of the accumulation yield of PLA to 11% DCW has been achieved after an extensive work of metabolic engineering (Jung et al., 2010). In this study, we report for the first time the production of PDLA in an eukaryotic organism, the yeast *Y. lipolytica* and a 2.6% DCW accumulation of PLA was obtained after limited modifications (the deletion of Yl*DLD1* responsible of the D-lactate consumption and the expression of the two enzymes of the PLA biosynthesis pathway).

More importantly, our results give valuable insights for PDLA production in compartmentalized eukaryotes. We investigated three cellular compartments (cytosol, mitochondria and peroxisomes) for the two steps of the PDLA production pathway. Mitochondria present the advantage of a high acetyl-CoA pool (Xua et al., 2016), which is essential as it is the co-substrate of the reaction for the CoA transferase activity of the PCT, whereas peroxisomes have already been proven to be a good environment for PHA production (Rigouin et al., 2019). It is interesting to note that the best PDLA production is obtained when the two enzymes of the pathway are located in different cellular compartments. Indeed, the production of the first intermediate, D-lactyl-CoA, is optimal when the PCT is expressed in the cytosol, and its polymerization in PDLA, only occurs when the PHA synthase is addressed to the peroxisomes. Our results clearly demonstrate that metabolic pathway efficiency can be highly modified using enzyme targeting strategies.

In our particular case, lactyl-CoA was not produced when the PCT was targeted to the mitochondria. This result suggests that either the physiological environment of this organelle may inactivate the enzyme, or that D-lactic acid may not be transported in the mitochondria in this condition. When the PCT is targeted to peroxisomes, a small production of lactyl-CoA has been detected, which indicates that the enzyme is active in that compartment to some extent. However, we could not exclude an incomplete transport of the PCT into the peroxisome via the PEX5 import pathway inducing some residual activity in the cytosol. Nevertheless, the lactyl-CoA production is much lower into the peroxisome than the one observed in the cytosol. One possible explanation could be that the deletion of the β-oxidation pathway (necessary for the production of a homopolymer deprived of any hydroxyalkanoates) severely reduced the acetyl-CoA pool and thus greatly limits the PCT activity in this compartment.

Interestingly, the highest PDLA production is achieved when the PHA synthase is targeted to peroxisomes. It suggests that the D-lactyl-CoA is efficiently transported to peroxisomes where it can be polymerized. This could be done by half-ABC peroxisomal CoA transporters likely Pxa1p or Pxa2p (Dulermo et al., 2015). We strongly suspect that the confined environment of the peroxisome helps to overcome the low affinity of the enzyme for D-lactyl-CoA by bringing the reaction partners closer and by increasing their local concentration. This is also supported by previous reports showing that dimerization of this enzyme is crucial for its activity (Wodzinska et al., 1996; Zhang et al., 2000; Chek et al., 2020). This spatial constraint of the peroxisome is also likely to help the PHA synthase to stay attached to the polymer to ensure the elongation reaction, thus explaining the high molecular weight of PLA obtained here. The inheritance of peroxisome during cell division is another advantage of the system (Chang et al., 2007). Indeed, commonly, these organelles are not made *de novo*, but are distributed equitably between daughter and mother cells, thus ensuring a low polydispersity for the polymer.

The engineered yeast strain yielded a polymer solely composed of lactic acid without any 3-hydroxy-fatty acid contamination. This purity results from (1) confinement of the polymerization in peroxisomes with the use of a strain devoid of the beta oxidation pathway, meaning the absence of 3-hydroxy-fatty acid production in this compartment and, (2) PHA synthase has no access to 3-hydroxy-fatty acid which may be produced from *de novo* fatty acid synthesis as this one occurs in cytosol. The use of a eukaryotic host that allows compartmentalization is thus a major advantage to obtain a polymer only composed of lactic acid.

Nowadays, the synthesis of PLA is based on a biosourced synthon polymerized by a chemical process. The development of microorganism able to polymerize lactic acid opens the way to establish a fully biological process for plastic synthesis. With a biological polymerization, the downstream purification steps of lactic acid needed for the chemical process could be drastically reduced as we demonstrate that neither enantiopure nor highly concentrated lactic acid is needed to produce a polymer of excellent enantiopurity, which is a crucial characteristic for the polymer properties. To go further, both the biological lactic acid production and lactic acid polymerization could be combined in a one–pot bioreactor either by co-culturing strains dedicated for each step or by integrating the two steps in one unique organism. Indeed, in *E. coli*, the highest accumulation was reported for strains metabolically engineered to produce lactate. Such a strategy presents the benefit to alleviate limitations related with the import of the synthon into the cells. It could thus be interesting to metabolically engineer a PDLA producing strain to produce its own D-lactic acid or, conversely, to engineer metabolic pathway for PDLA in an eukaryotic chassis capable of D-lactic acid production. In this context, D-lactic acid eukaryotic producer such as *S. cerevisiae*, *Pichia kudriavzevii* or *Rhizopus oryzae* (Sauer et al., 2010; Baek et al., 2016; Park et al., 2018) could be good candidates. However, biological polymer production raise the question of its extraction, as the purification of microbial biopolymers is one of the major challenges to overcome for their industrial development (Kosseva and Rusbandi, 2018). In this field, the polymer extraction could benefit of the recent advances on lipid extraction developed in *Y. lipolytica* (Drévillon et al., 2018; Yook et al., 2019).

## Data Availability Statement

The raw data supporting the conclusions of this article will be made available by the authors, without undue reservation, to any qualified researcher.

## Author Contributions

JV realized the *in silico* sequence analysis. SL, SD, JV, and MB carried out strain constructions and monitoring of the culture. SL realized the polymer extractions and analysis by gel permeation chromatography. VB setup and performed the NMR analysis. MF and FBe set up the condition for CoA extraction and analysis by GC-MS. SL, SD, JV, MB, CR, and ZG performed the experiments. All participated to the analysis of the results. SL, FBo, AM, and J-MN contributed to the design of the study. FBo, AM, and J-MN supervised the study. SL wrote the first draft of the manuscript. All authors contributed to manuscript revision, read and approved the submitted version.

## Conflict of Interest

The authors declare that this project was co-funded by national funding and the Carbios company. The funder Carbios had the following involvement in the study: co-design and co-supervision of the study and contribution to manuscript redaction. AM is employed by the Carbios company. Some of the results are part of a patent corresponding to application number WO2017/10577 with SL, SD, JV, VB, J-MN, AM and FB as co-inventors. The remaining authors declare that the research was conducted in the absence of any commercial or financial relationships that could be construed as a potential conflict of interest.

## Footnotes

1 www.ncbi.nlm.nih.gov

2 http://www.uniprot.org/help/uniprotkb

3 http://gryc.inra.fr/

4 http://www.ebi.ac.uk/Tools/msa/clustalo/

5 http://pfam.xfam.org/

6 http://www.cbs.dtu.dk/services/

7 https://ihg.gsf.de/ihg/mitoprot.html

## Abbreviations

CoA: coenzyme A
LDH: lactate dehydrogenase
PCT: propionyl-CoA transferase
PDLA: poly-D-lactic acid
PHA: polyhydroxyalkanoic acid
PLA: polylactic acid
PLLA: poly-L-lactic acid.
