# Supplementary material for: Engineering the Yeast Yarrowia lipolytica for Production of Polylactic Acid Homopolymer
Source: Front Bioeng Biotechnol. 2020 Oct 22;8:954. doi: 10.3389/fbioe.2020.00954 (PMC7609957; doi:10.3389/fbioe.2020.00954)
Supplement: Supplementary file 1 [file Data_Sheet_1.pdf]

Supplementary Material

for

Engineering the yeast *Yarrowia lipolytica* for production of polylactic acid homopolymer

Sophie Lajus, Simon Dusséaux, Jonathan Verbeke, Coraline Rigouin, Zhongpeng Guo, Maria Fatarova, Floriant Bellvert, Vinciane Borsenberger, Mélusine Bressy, Jean-Marc Nicaud, Alain Marty, Florence Bordes

Supplementary Data :Nucleotide sequence of optimized genes

Figure S1: Metabolic process for PDLA production *in vivo*.

Figure S2: Quantification of acetyl-CoA produced in strain expressing PCT protein from different organisms.

Figure S3: Quantification of acetyl-CoA produced in strain expressing PCT protein from *C. propionicum* targeted to different subcellular compartments.

Figure S4: PDLA accumulation visualized in microscopy.

Figure S5: PDLA quantification with different D-lactic acid initial concentration.

Figure S6: Quantification of acetyl-CoA produced in strain expressing *C. propionicum* cytosolic PCT protein under the control of different promotors.

Figure S7: PDLA quantification in strains expressing PCT and PHA polymerase proteins under the control of different promotor.

Table S1: Primers for cassette construction and strain verification

Table S2: Primers for site directed mutagenesis

1 Supplementary Data :Nucleotide sequence of optimized genes

CpPCT:

ATGCGAAAGGTTCCCATCATCACTGCTGACGAGGCTGCCAAGCTCATCAAGGACGGAGATACCGTTA
CTACTTCGGGTTTTGTCGGAAACGCTATCCCTGAGGCTCTGGACCGAGCTGTCGAGAAGCGATTCCT
CGAGACCGGCGAGCCTAAGAACATTACTTACGTTTACTGTGGATCTCAGGGTAACCGAGACGGACGA
GGTGCTGAGCACTTTGCCCATGAGGGCCTGCTCAAGCGATACATTGCTGGACACTGGGCCACCGTTC
CCGCTCTGGGAAAGATGGCCATGGAGAACAAGATGGAGGCTTACAACGTGTCCCAGGGAGCCCTGTG
CCACCTCTTCCGAGACATCGCCTCGCATAAGCCCGGTGTTTTCACCAAGGTCGGCATCGGAACTTTT
ATTGACCCTCGAAACGGCGGCGGCAAGGTCAACGACATCACCAAGGAAGACATTGTTGAGCTGGTGG
AGATTAAGGGCCAGGAGTACCTCTTCTACCCCGCCTTTCCTATCCACGTGGCTCTGATTCGAGGAAC
CTACGCCGACGAGTCCGGTAACATCACTTTTGAGAAGGAAGCCGCTCCCCTCGAGGGAACCTCTGTC
TGTCAGGCTGTTAAGAACTCCGGCGGAATTGTGGTCGTTCAGGTCGAGCGAGTGGTCAAGGCCGGAA
CTCTGGACCCCCGACATGTCAAGGTTCCTGGTATCTACGTGGATTACGTTGTGGTCGCTGACCCCGA
GGATCACCAGCAGTCGCTGGACTGCGAGTACGATCCCGCCCTCTCTGGCGAGCATCGACGACCTGAG
GTTGTGGGAGAGCCCCTGCCTCTCTCGGCTAAGAAGGTCATCGGCCGACGAGGAGCCATTGAGCTGG
AGAAGGACGTGGCTGTCAACCTCGGTGTGGGAGCTCCTGAGTACGTGGCTTCTGTTGCTGACGAGGA
AGGCATCGTCGATTTCATGACCCTGACTGCCGAGTCTGGAGCTATTGGTGGCGTGCCTGCTGGAGGT
GTCCGATTCGGTGCCTCCTACAACGCCGACGCTCTGATTGATCAGGGCTACCAGTTTGACTACTACG
ATGGCGGAGGTCTGGACCTCTGTTACCTGGGTCTCGCTGAGTGCGATGAGAAGGGCAACATCAACGT
GTCCCGATTCGGTCCCCGAATTGCCGGCTGTGGCGGCTTCATCAACATTACCCAGAACACTCCTAAG
GTTTTCTTTTGCGGCACCTTCACTGCTGGTGGCCTGAAGGTGAAGATCGAGGACGGCAAGGTCATCA
TTGTCCAGGAAGGCAAGCAGAAGAAGTTCCTGAAGGCCGTCGAGCAGATCACCTTTAACGGAGACGT
TGCCCTCGCTAACAAGCAGCAGGTGACCTACATTACTGAGCGATGTGTCTTCCTGCTCAAGGAAGAC
GGTCTGCACCTCTCTGAGATTGCTCCTGGCATTGATCTGCAGACCCAGATCCTCGACGTGATGGATT
TTGCTCCTATCATTGACCGAGATGCCAACGGCCAGATTAAGCTGATGGATGCTGCTCTGTTTGCTGA
GGGTCTGATGGGCCTGAAGGAGATGAAGTCTTAA

EnPCT:

ATGACCCACCCCCAGCAGGCCGTTCACGCCGCTTCGCTCCAGAACCCCGAGGCTTTTTGGTCCCATC
ACGCCCAGCAGCTCCATTGGCACAAGAAGCCCTCGCGAGCCATTGGCCGATCTACCAAGACTCTGGC
TTCTGGAGCCTCCCACGAGTCCTGGTCGTGGTTCCCTGACGGAGAGATCTCCACCACTTACAACTGT
GTGGATCGACATGTCCTGAACGGCAACGGAGACAACGTGGCCATCATTTGGGATTCTGCTGTCACCG
GCAAGAAGGAGAAGTACACTTACCGACAGCTGCTCGACGAGGTCGAGGTTCTGGCTGGTGTCCTCCG
AGAGGAGGGCGTTAAGAAGGGAGACGTGGTCATCATCTACATGCCCATGATCCCTGCCGCTCTGATT
GGAGCTCTCGCTGTCGCTCGACTGGGTGCTATTCACGCCGCTGTTTTCGGCGGATTTGCCGCTAAGT
CCCTGGCTCAGCGAATTGAGGCTGCTCGACCCCGAGCTATCCTCACCGCTTCTTGCGGTATTGAGGG
CGCCAAGGGACCCATCGCTTACCGACCTCTGGTGGAGGGCGCTATTGAGGCCTCTTCCTTCAAGCCC
GAGAAGGTCCTGATCTGGCAGCGAGACCAGCTCCGATGGAACAACCCTGATAAGCTGGGTGGCCAGC
GAAACTGGAACCGACTCGTGAAGTCCGCCCGAATGCGAGGCATTCGAGCTGAGCCCGTGCCTGTCCG
ATCTACCGACGGACTGTACATCATCTACACTTCCGGTACCACTGGCCTCCCCAAGGGAGTTGTGCGA
GAGGCCGGAGGTCACGCTGTGGGTCTGTCTCTCTCCATCAAGTACCTGTTCGACATTCATGGTCCCG
GCGATACCATGTTTTGTGCCTCCGACATTGGTTGGGTCGTTGGCCACTCGTACATCCTGTACGCCCC
TCTGCTCGTCGGAGCTACCACTGTTCTCTTCGAGGGAAAGCCTGTGGGTACCCCTGACGCTGGTACT
TTTTGGCGAGTGGTCGCCGAGCATAAGGCTAACGTCCTGTTCACCGCTCCCACTGCCCTCCGAGCTA
TTCGAAAGGAGGACCCTGATAACAAGCACTTTGAGAAGGTGGCCGGTGACAACAACCTGCGACATCT
CCGAGCCCTGTTCCTCGCTGGCGAGCGATCGGAGCCCTCTATCGTCCGAGCCTACCAGGACCTGCTC
ACCAAGCACGCCGCTCGAGGAGCTCTGGTTGTGGATAACTGGTGGTCGTCTGAGTCGGGCTCTCCTA
TTTCCGGACTGGCTCTCCGATCGGCTGTCGGTCGAGTTCCTCCTCGATCGGACGAGTACGATGTGGC
CCCCCTGGCTATCCGACCTGGATCTGCCGGTCTCCCCATGCCTGGTTTCGACGTCCGAGTCGTTGAC
GATGAGGGCAACGAGGTTGCCCAGGGCACCATGGGAAACATTGTGATGGCTACTCCCCTGGCCCCTA
CCGCTTTCACTCGACTCTTTAACGACGATGAGCGATTCTACAAGGGATACCTGAAGCGATTTGGCGG
ACGATGGCTCGACACCGGCGACGCTGGTATGATCGACCAGGATGGCTACATTCACGTGATGTCCCGA
TCGGACGATATCATTAACGTCGCCGCTCACCGATTCTCTACTGGACAGGGTTCCATCGAGCAGGCCA
TTCTGTCGCACCCCGCCATTGGAGAGGCTTCTGTGGTCGGCATCCCCGACGCCCTGAAGGGACATCT
CCCTTTCGCTTTTATCACCCTGAAGCAGTCCGGTGGTAACTCGCCTGCTCGACCTTCTGCTGAGCTG
TTCAACTCCGTTAACCGACTCGTGCGAGAGCAGATCGGAGCTATTGCCTCCCTGGGAGGAATGATCC
AGGGCCAGGGAATGATTCCCAAGACCCGATCTGGCAAGACTCTCCGACGAGTGCTGCGAGAGCTCGT
CGAGAACGGAGCCCGAGGTGAGTTCGAGAAGGAGGTTGCTGTGCCTCCTACCGTGGAGGACCGAGGC
GTTGTGGAGGTTGCCCGAGAGAAGGTGCGAGAGTACTTCGAGTCTCAGTCCGGATCGCCCAAGGCTA
AGCTGTAA

EcPCT:

ATGAAACCTGTCAAACCGCCTCGAATCAACGGCCGAGTTCCAGTTCTCTCTGCCCAGGAAGCCGTTA
ACTACATTCCCGATGAGGCTACCCTCTGTGTCCTTGGCGCTGGAGGAGGCATTCTTGAGGCCACCAC
GCTGATTACAGCCCTGGCTGACAAGTACAAGCAGACGCAGACTCCCCGAAATCTGTCCATTATCTCT
CCCACAGGACTTGGTGATCGAGCTGATCGAGGCATTTCCCCTCTGGCACAAGAGGGACTGGTGAAGT
GGGCGCTGTGCGGTCATTGGGGCCAGTCTCCACGAATTAGCGATCTGGCCGAACAGAACAAGATTAT
TGCCTACAACTACCCTCAGGGTGTGCTTACCCAGACCCTCCGAGCCGCAGCTGCCCATCAACCCGGC
ATTATCTCCGACATCGGCATTGGAACCTTTGTCGATCCCCGACAGCAGGGCGGCAAGCTGAACGAGG
TGACCAAAGAGGACCTCATCAAGTTGGTTGAGTTCGACAACAAGGAGTACCTTTACTACAAGGCCAT
TGCTCCCGATATTGCCTTCATTCGTGCAACCACCTGCGATTCCGAAGGCTACGCCACTTTTGAGGAC
GAGGTGATGTATCTCGACGCCCTGGTTATTGCGCAAGCTGTCCACAACAACGGTGGAATCGTGATGA
TGCAGGTCCAGAAGATGGTTAAGAAGGCCACGCTTCACCCCAAGTCCGTGCGTATCCCCGGTTACCT
CGTCGACATCGTGGTCGTTGACCCGGATCAGTCTCAGTTGTATGGTGGCGCCCCAGTCAACCGATTC
ATCTCTGGCGACTTCACCCTCGACGACTCCACCAAGCTGTCGCTTCCCCTCAATCAGCGGAAGCTTG
TCGCTAGACGAGCACTGTTTGAGATGCGGAAAGGAGCGGTCGGAAACGTGGGTGTCGGCATTGCCGA
TGGTATCGGACTCGTTGCCCGAGAAGAAGGTTGTGCTGACGACTTCATTTTGACCGTCGAGACTGGC
CCTATCGGCGGAATCACTTCGCAAGGAATCGCCTTTGGCGCCAATGTCAACACCCGAGCCATCCTTG
ACATGACGTCCCAGTTTGACTTCTACCACGGAGGAGGTCTGGACGTGTGCTACCTGTCGTTTGCAGA
AGTCGACCAGCATGGCAACGTTGGTGTCCACAAGTTCAACGGCAAGATCATGGGAACCGGAGGCTTC
ATCGACATCTCCGCTACTTCCAAGAAGATCATCTTCTGTGGCACACTCACCGCTGGTTCTCTCAAGA
CTGAGATTGCTGACGGTAAGCTGAACATTGTGCAGGAGGGCCGAGTCAAGAAGTTCATCCGAGAACT
GCCTGAGATCACCTTCAGCGGCAAGATCGCCCTGGAGAGAGGTCTGGATGTGCGGTACATCACAGAG
AGAGCTGTGTTTACTCTGAAAGAGGATGGTCTGCACTTGATCGAGATTGCTCCTGGTGTTGACCTGC
AGAAGGACATCCTCGACAAGATGGATTTCACTCCCGTGATCTCCCCTGAGCTGAAGCTGATGGACGA
GCGACTCTTCATTGACGCTGCCATGGGTTTTGTCCTCCCCGAGGCTGCGCACTAA

RePCT:

ATGAAGGTGATTACCGCCAGAGAAGCAGCGGCTCTTGTGCAGGACGGTTGGACTGTTGCATCGGCTG
GATTCGTTGGCGCAGGCCATGCTGAGGCAGTCACCGAAGCCCTTGAGCAGCGATTTCTGCAATCGGG
TCTGCCACGAGATCTGACCCTCGTCTACTCTGCTGGACAGGGCGATCGTGGTGCCCGAGGTGTGAAC
CACTTCGGCAATGCCGGCATGACCGCCAGCATCGTCGGCGGCCATTGGAGATCCGCAACCAGACTCG
CCACCCTGGCCATGGCTGAGCAGTGTGAGGGCTACAACCTGCCTCAAGGCGTCCTTACGCACCTGTA
CCGAGCCATTGCTGGCGGTAAACCTGGTGTCATGACCAAGATCGGCCTCCATACGTTCGTCGACCCA
CGAACCGCCCAAGATGCCCGATACCATGGCGGCGCGGTTAACGAGCGAGCACGGCAGGCCATTGCCG
AGGGAAAGGCTTGCTGGGTTGACGCCGTGGACTTTCGAGGCGATGAGTACCTGTTCTACCCCTCGTT
TCCCATCCACTGTGCGCTCATTCGGTGCACTGCCGCTGACGCCCGAGGAAACCTCTCCACTCACAGA
GAGGCCTTTCACCACGAACTTTTGGCAATGGCCCAAGCTGCTCACAACTCCGGAGGCATCGTCATCG
CGCAGGTGGAGTCCCTCGTGGACCACCACGAGATTCTGCAGGCCATCCACGTTCCAGGCATTCTGGT
GGACTACGTCGTGGTTTGCGACAACCCCGCTAATCACCAGATGACCTTCGCCGAGTCCTACAACCCT
GCGTACGTCACGCCTTGGCAGGGAGAAGCTGCCGTGGCCGAAGCCGAGGCCGCTCCCGTCGCTGCTG
GACCCCTTGACGCGCGGACCATCGTGCAGCGTCGAGCCGTTATGGAGCTGGCCCGACGAGCCCCGCG
AGTTGTGAACCTCGGTGTCGGAATGCCTGCTGCCGTTGGTATGCTCGCCCATCAGGCTGGACTCGAC
GGCTTCACCCTGACTGTGGAGGCAGGCCCCATTGGTGGTACTCCCGCTGACGGACTGTCCTTTGGTG
CCTCTGCTTATCCGGAGGCTGTCGTCGACCAGCCTGCCCAGTTCGACTTCTACGAAGGCGGTGGCAT
TGACCTTGCCATCCTCGGCTTGGCTGAGCTCGATGGTCACGGCAACGTCAACGTGTCCAAGTTCGGT
GAGGGAGAAGGAGCCTCCATTGCTGGTGTTGGCGGTTTCATCAACATCACCCAGTCTGCTCGAGCCG
TCGTGTTCATGGGAACACTGACAGCAGGTGGACTTGAGGTTCGAGCTGGTGATGGAGGACTCCAGAT
CGTCCGAGAGGGCCGAGTCAAGAAGATCGTCCCTGAGGTGTCTCACCTGTCCTTTAACGGTCCCTAT
GTGGCTTCTCTCGGAATCCCTGTCCTGTACATCACTGAGCGAGCTGTTTTCGAGATGCGAGCTGGAG
CTGATGGCGAAGCCCGATTGACTCTGGTGGAGATTGCGCCCGGTGTCGACCTTCAGCGGGACGTTTT
GGACCAGTGTAGCACACCCATTGCTGTCGCCCAGGATCTGCGTGAGATGGATGCCCGTCTGTTTCAG
GCCGGTCCCCTGCATCTGTAA

ACS2:

ATGTCGGAGGACCACCCTGCTATTCACCCCCCTTCTGAGTTCAAGGACAACCACCCCCACTTCGGCG
GCCCCCATCTCGACTGCCTCCAGGACTACCACCAGCTGCATAAGGAGTCTATCGAGGACCCCAAGGC
TTTCTGGAAGAAGATGGCCAACGAGCTGATTTCCTGGTCGACCCCTTTCGAGACTGTGCGATCGGGC
GGATTTGAGCACGGTGACGTCGCTTGGTTCCCTGAGGGTCAGCTGAACGCTTCTTACAACTGTGTCG
ACCGACATGCCTTTGCTAACCCCGATAAGCCTGCTATCATTTTCGAGGCCGACGAGCCTGGCCAGGG
ACGAATCGTGACCTACGGAGAGCTGCTCCGACAGGTGTCTCAGGTCGCCGCTACCCTGCGATCCTTC
GGCGTCCAGAAGGGAGACACTGTTGCCGTGTACCTCCCCATGATCCCTGAGGCTATTGTGACCCTGC
TCGCCATCACTCGAATTGGCGCTGTTCACTCCGTGATCTTCGCTGGTTTTTCTTCCGGCTCGCTGCG
AGACCGAATTAACGATGCCAAGTCGAAGGTGGTCGTTACCACTGACGCTTCTATGCGAGGTGGCAAG
ACCATCGACACTAAGAAGATTGTCGACGAGGCCCTCCGAGATTGCCCCTCTGTGACCCATACTCTGG
TCTTTCGACGAGCCGGAGTGGAGAACCTCGCTTGGACCGAGGGTCGAGACTTCTGGTGGCACGAGGA
GGTGGTCAAGCATCGACCCTACCTGGCTCCTGTCCCTGTTGCTTCCGAGGACCCTATTTTCCTGCTC
TACACCTCTGGCTCCACCGGAACTCCTAAGGGTCTGGCCCACGCTACCGGAGGTTACCTGCTCGGAG
CCGCTCTCACTGCCAAGTACGTGTTTGACATCCACGGTGACGATAAGCTGTTCACCGCCGGCGACGT
CGGTTGGATTACCGGTCATACTTACGTTCTCTACGGACCCCTGATGCTCGGTGCCACCACTGTTGTG
TTTGAGGGAACCCCCGCTTACCCTTCGTTCTCTCGATACTGGGACATCGTTGACGATCACAAGATTA
CCCATTTTTACGTGGCCCCCACTGCTCTGCGACTGCTCAAGCGAGCCGGCACCCACCATATCAAGCA
CGACCTGTCGTCTCTCCGAACTCTGGGATCTGTGGGAGAGCCCATTGCCCCTGACGTCTGGCAGTGG
TACAACGATAACATCGGTCGAGGCAAGGCTCACATTTGTGACACCTACTGGCAGACCGAGACTGGCT
CTCATATCATTGCCCCCATGGCTGGAGTCACCCCCACTAAGCCTGGTTCCGCCTCGCTGCCTGTGTT
CGGCATCGACCCCGTTATCATTGATCCTGTGTCGGGCGAGGAGCTCAAGGGAAACAACGTCGAGGGT
GTTCTGGCTCTCCGATCGCCTTGGCCTTCTATGGCTCGAACCGTGTGGAACACTCACGAGCGATACA
TGGAGACCTACCTGCGACCCTACCCTGGATACTACTTTACTGGAGACGGTGCCGCTCGAGACAACGA
TGGCTTCTACTGGATTCGAGGACGAGTCGACGATGTCGTTAACGTTTCCGGCCATCGACTCTCGACC
GCCGAGATCGAGGCCGCTCTGATTGAGCACGCCCAGGTTTCTGAGTCCGCTGTGGTCGGCGTGCATG
ACGATCTGACCGGACAGGCCGTCAACGCTTTTGTTGCCCTCAAGAACCCCGTGGAGGACGTCGATGC
CCTCCGAAAGGAGCTGGTTGTGCAGGTCCGAAAGACCATCGGACCCTTCGCCGCTCCTAAGAACGTT
ATCATTGTGGACGATCTGCCCAAGACTCGATCCGGCAAGATCATGCGACGAATTCTCCGAAAGGTGC
TGGCTGGAGAGGAGGACCAGCTCGGCGATATTTCTACCCTGGCTAACCCTGACGTCGTTCAGACTAT
CATTGAGGTGGTCCACTCCCTGAAGAAGATGGGTGCCGGTGTCACTGAGGACCAGTTCAAGTCCAAG
CTGTAA

2 Supplementary Figures and Tables

2.1 Supplementary Figures

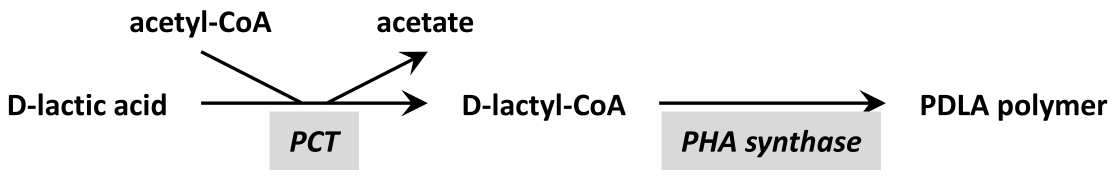

Figure S1: Metabolic process for PDLA production *in vivo*.

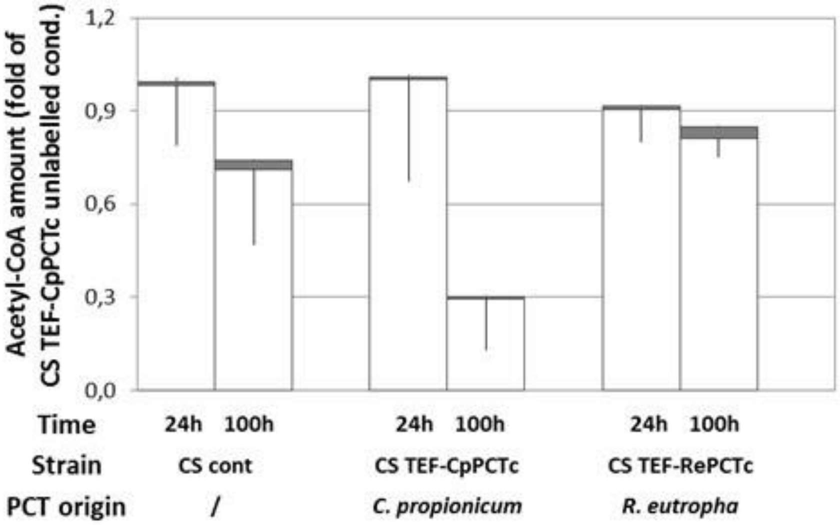

Figure S2: Quantification of acetyl-CoA produced in strain expressing PCT protein from different organisms.

Strains were grown for 24 hours and 100 hours on minimum medium containing a mixture of 5g/L of unlabelled L-lactic acid and 5g/L of labelled D-lactic acid 3-^{13}C. After a normalization, results were expressed as fold of CS + TEF-CpPCTc strain unlabeled result at 24 hours. Only strains showing a detectable level of lactyl-CoA have been represented. White bars: unlabeled compound, grey bars: ^{13}C-labeled compound. For clarity purposes, only half error bars have been represented; to the bottom for unlabelled compound and to the top for labelled one. N=3 independent experiments.

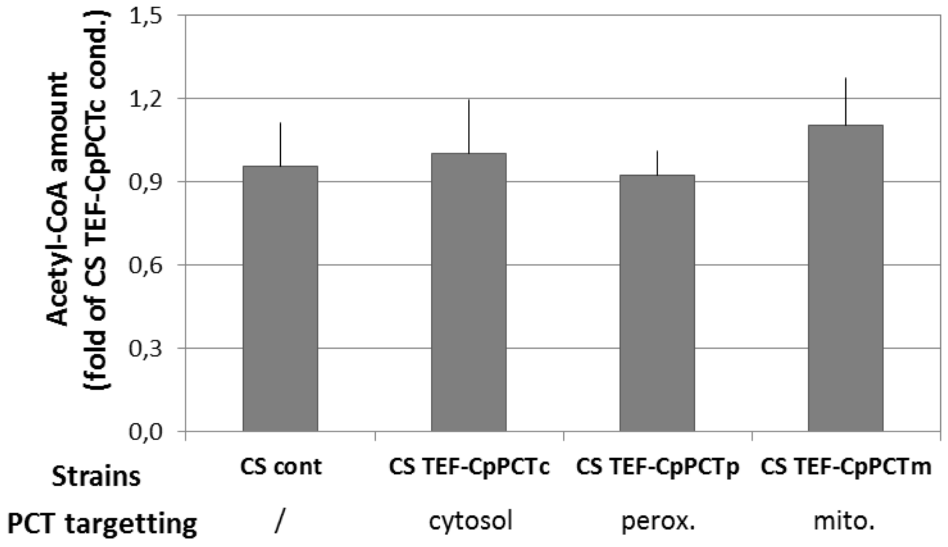

Figure S3: Quantification of acetyl-CoA produced in strain expressing PCT protein from *C. propionicum* targeted to different subcellular compartments.
Strains were grown for 24 hours on minimum medium containing a mixture of 10 g/L of lactic acid. After a normalization, results were expressed as fold of CS TEF-CpPCTc strain result. For clarity purposes, only half error bars have been represented. N=3 independent experiments.

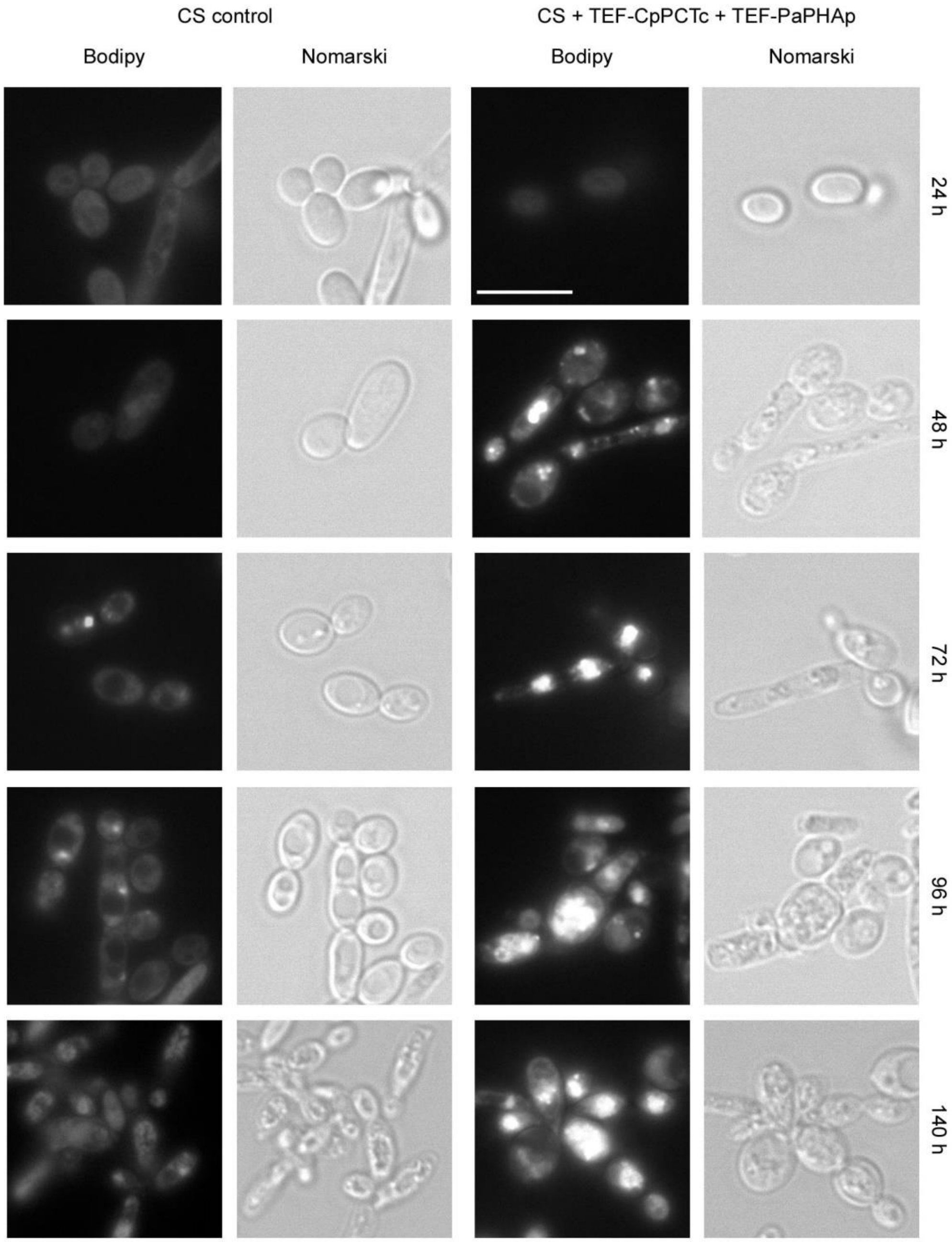

Figure S4: PDLA accumulation visualized in microscopy.
CS Control strain (left part) and CS + TEF-CpPCTc + TEF-PaPHAp (right part) were grown for 5 days on minimum medium containing a mixture of 10 g/L of lactic acid. PDLA was observed on living cells using Bodipy fluorescent dye at different times. Bar = 10µm

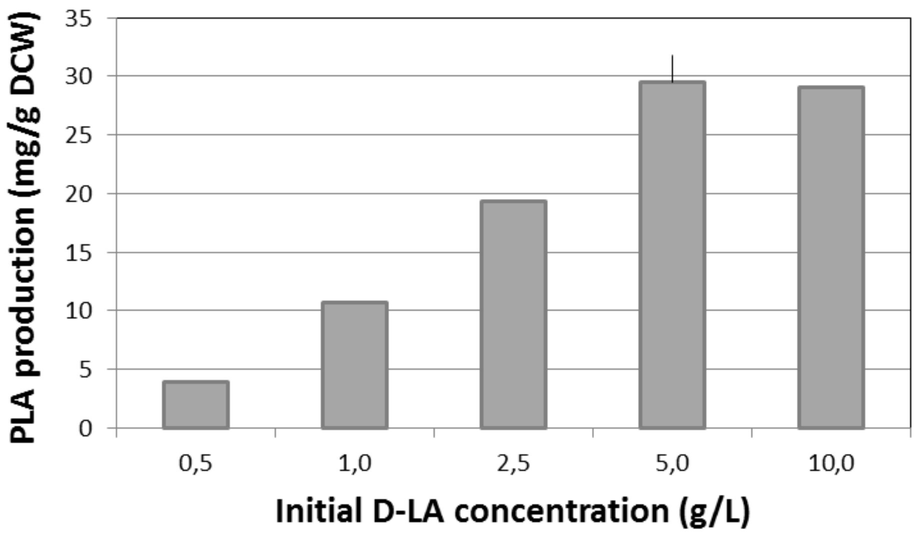

Figure S5: PDLA quantification with different D-lactic acid initial concentration.
Strains expressing *C. propionicum* cytosolic PCT and *P. aeruginosa* peroxisomal quadruple PHA synthase variant were grown for 5 days on minimum medium containing different initial concentration of D-lactic acid. After extraction, PLA quantification was measured by NMR using PLA specific peaks. N=1 to 3 independent experiments.

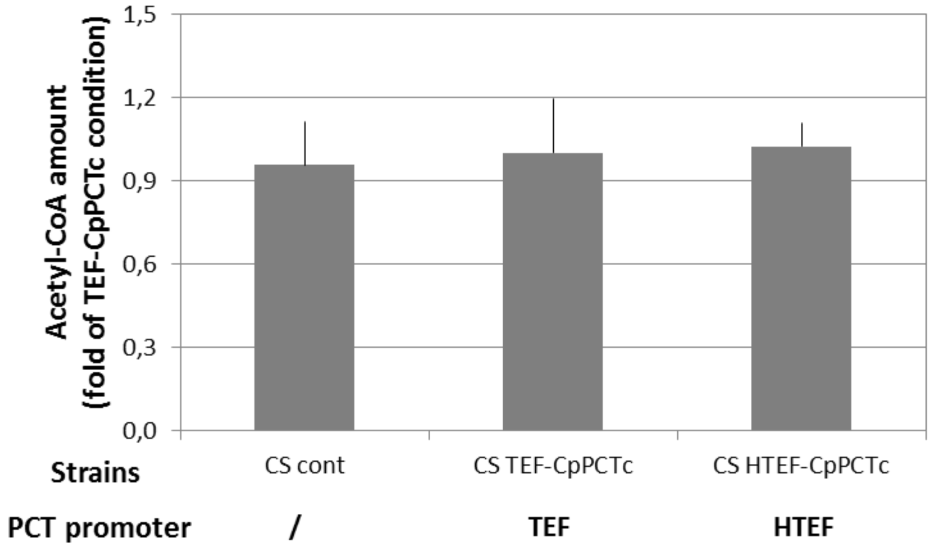

Figure S6: Quantification of acetyl-CoA produced in strain expressing *C. propionicum* cytosolic PCT protein under the control of different promotors.

Strains were grown for 24 hours on minimum medium containing a mixture of 10 g/L of lactic acid. After a normalization, results were expressed as fold of CS TEF-CpPCTc strain result. For clarity purposes, only half error bars have been represented. N=3 independent experiments.

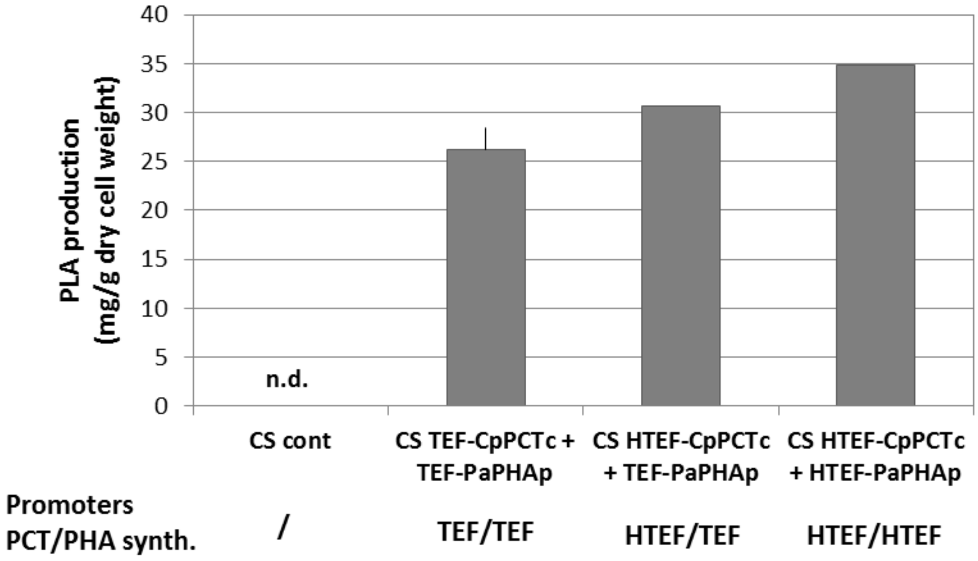

Figure S7: PDLA quantification in strains expressing PCT and PHA polymerase proteins under the control of different promotors.
Strains expressing proteins under the control of different promotors were grown for 5 days on minimum medium containing a mixture of 10 g/L of lactic acid. After extraction, PLA quantification was measured by NMR using PLA specific peaks. N=3 minimum independent experiments for the two first strains and N=1 for the last two. n.d., not detected.

2.2 Supplementary Tables

Table S1: Primers for cassette construction and strain verification

Name	Sequence	Site inserted
ylDLD1for	CACACATCGGATCCCACAATGAACAGAATCTCACGAGCCGCCCAGAGC	*Bam* HI
ylDLD1rev	GAGACTTTCCTAGGTTAAGTCTTACCAGCCTTGTTGTTATCCAGAGTCTTGAGAC	*Avr* II
ylCYB21for	CACTCACATGATCACACAATGCCTATCACCCAGAAGATTCTGTCGCTCAG	*Bcl* I
ylCYB21rev	CTTCTGGGTCCTAGGTAAAGCTTGACGATGGCGGGTCCGATTCGAAC	*Avr* II
ylDLD1P1	GAACTGCTCCTGTGAATCTCTTAACGAACACAGTCG	/
ylDLD1P2	CCTCGCTACCTTAGGACCGTTATAGTTACGATTACCCTGTTATCCCTACCGGCGAAACTGGATGTGTGAATGTATCAAGAATGGAAAGAGAC	I-*Sce* I
ylDLD1T1	GGTAGGGATAACAGGGTAATCGTAACTATAACGGTCCTAAGGTAGCGAGGACCTGCAAAGTCTCTCACGTACGTCTGTAATGATACAAAATG	I-*Sce* I
ylDLD1T2	CTGGGCCAGGCGAATATTCTCACCACCAG	/
ylCYB21P1	GATTGGCCAGCAGCCAGGCCGCC	/
ylCYB21P2	CGATTACCCTGTTATCCCTACCGAGTCGCCAAAGGTGGTGAATGGACGGAGTCGCCAAAGGTGGTGAATGGACG	I-*Sce* I
ylCYB21T1	GGTAGGGATAACAGGGTAATCGTAACTATAACGGTCCTAAGGTAGCGAGGGATCAACCGAAGCTGTCTGGTTCGAATCG	I-*Sce* I
ylCYB21T2	GACGCTCTGATAGAGCGGCGACGAGG	/
DLD1VER1	CAGAGGTCACTCGGAGGAGCCTGAAATC	/
DLD1VER2	GGAACTTTCGGTTCTCGAAGACCTTCTGTCC	/
CYB21VER1	CTGCATCTACGGATGTGCATATCTACTGTGC	/
CYB21VER2	GGTGTACATACGCCGAATAATGTCTCTGC	/
URA3sens	CGGCCAGCATGAGCAGACCTCTGGCCAG	/
LEU2sens	CGCTGTTGAGGCTGCCGTCAAGGAGTCCG	/

Table S2: Primers for site directed mutagenesis

Name	Sequence	Mutations
PaPHA_E130for	CAACCTGCTCACCGACGCTATGTCTCCCACC	E130D
PaPHA_E130rev	GGTGGGAGACATAGCGTCGGTGAGCAGGTTG	E130D
PaPHA_S325for	CCCAGCTGGTGACCGTGCTGGACTTTGAGC	S325T
PaPHA_S325rev	GCTCAAAGTCCAGCACGGTCACCAGCTGGG	S325T
PaPHA_S477/Q481for	CCTGTCCAACCGAGGCCACATCATGTCTATCCTGAACC	S477R and Q481M
PaPHA_S477/Q481rev	GGTTCAGGATAGACATGATGTGGCCTCGGTTGGACAGG	S477R and Q481M
